# Supplementary material for: Microfluidic dose–response platform to track the dynamics of drug response in single mycobacterial cells
Source: Sci Rep. 2022 Nov 15;12:19578. doi: 10.1038/s41598-022-24175-9 (PMC9666435; doi:10.1038/s41598-022-24175-9)
Supplement: Supplementary file 1 — Supplementary Figures. [file 41598_2022_24175_MOESM1_ESM.pdf]

Supplementary information

Microfluidic dose-response platform to track the dynamics of drug response in single mycobacterial cells

Maxime Mistretta,[1] Nicolas Gangneux[1], Giulia Manina[1,*]

[1]Institut Pasteur, Université de Paris, Microbial Individuality and Infection Laboratory, F-75015 Paris, France
*Correspondence: giulia.manina@pasteur.fr

Supplementary Figures and Legends

Supplementary Video Legends

Supplementary Video 1

Supplementary Video 2

Supplementary Video 3

Supplementary Table 1

Supplementary Data 1 to 7

Supplementary Figures and Legends

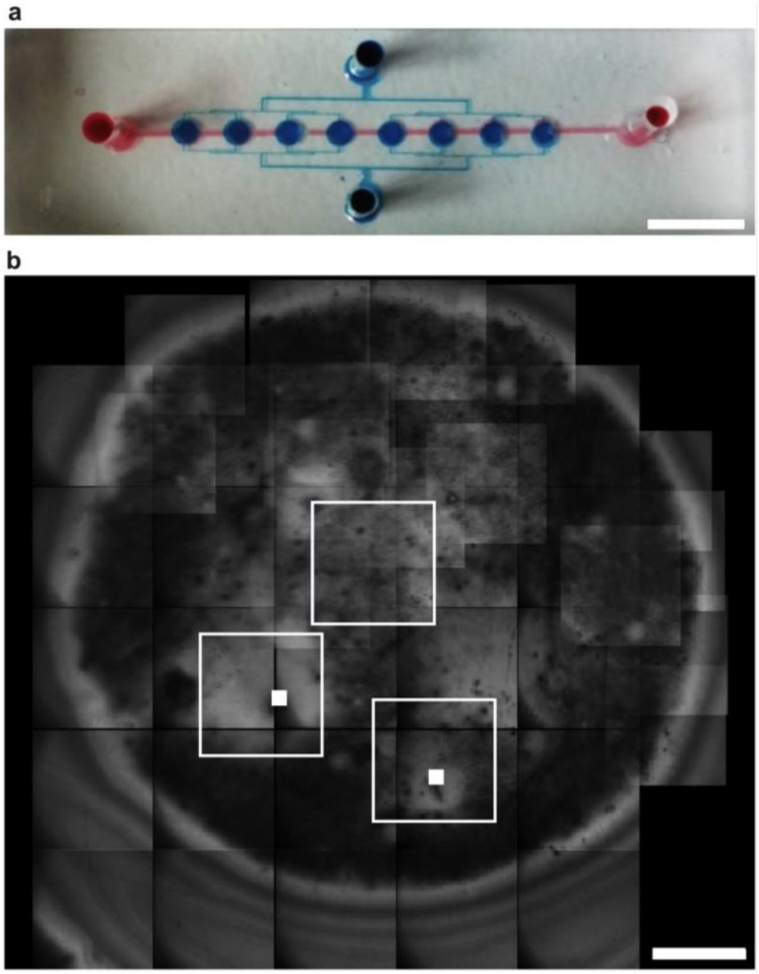

Supplementary Fig. 1

Single-condition prototype. a, Picture of a representative single-condition device, where eight microfluidic culture chambers are connected by a tree-shaped flow layer (FL, blue dye) and overlaid by the control layer (CL, red dye). Scale bar, 3.5 mm. **b,** Representative mosaic picture of the 2D-growth area formed between the PDMS membrane and the coverslip by applying a pressure of 25 mbar in the CL from the pressure controller. The field of view is excited at 475/28 nm and images are acquired in bright field. Scale bar, 50 µm. The brightest areas indicate the presence of a liquid interface (empty squares). *M. smegmatis* microcolonies are also marked (filled squares).

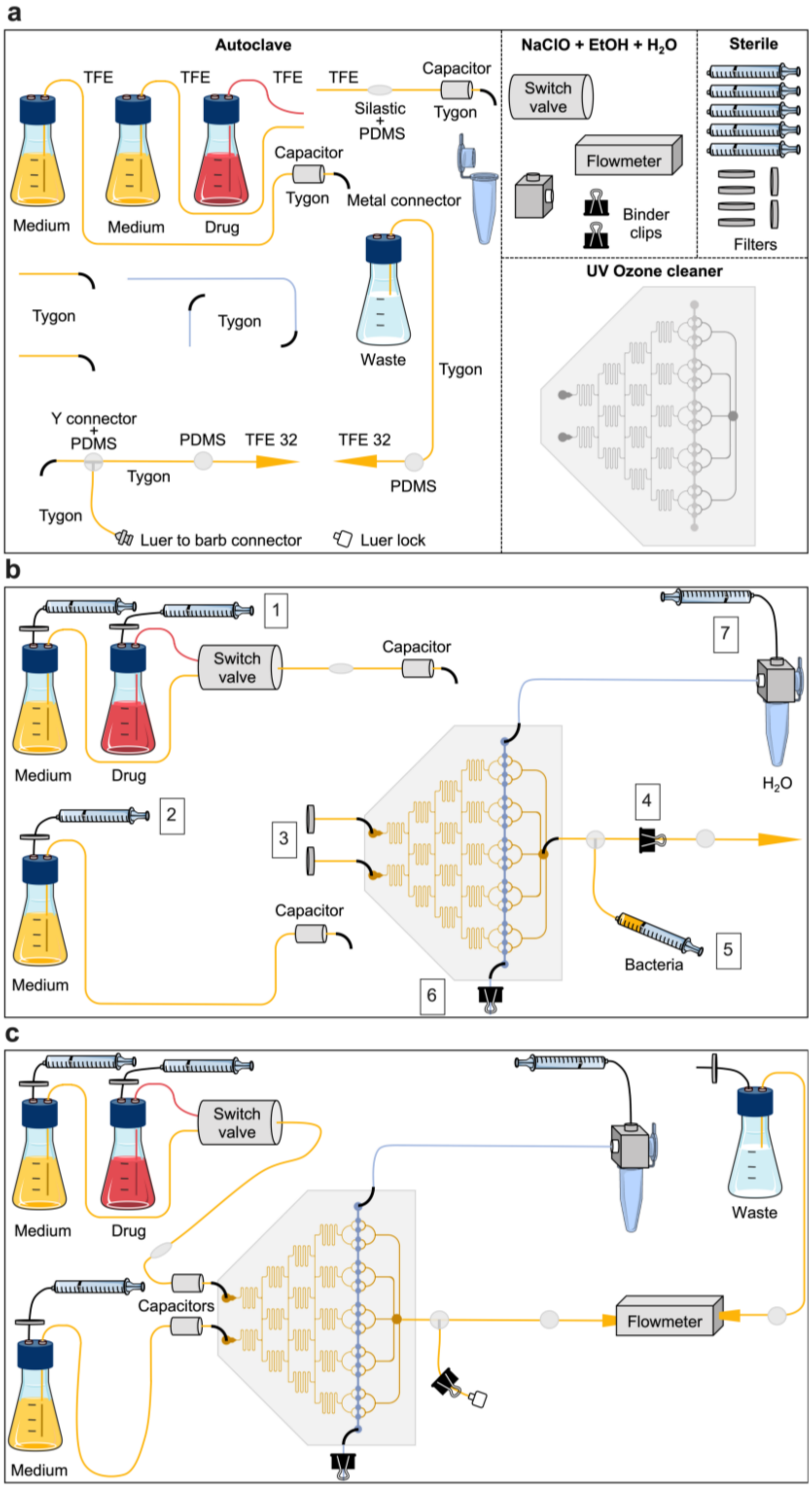

Supplementary Fig. 2

Preparation and assembly of the five-condition platform. a, Decontamination of the different components of the five-condition platform. The color of the tubing distinguishes the application: culture medium in the FL (yellow); culture medium containing the highest drug concentration in

the FL (red); water in the CL (blue). Materials constituting the different components are indicated. Cured PDMS is used to seal critical connections between tubing. Dashed lines separate different decontamination methods. Except for bottles, tubing and connectors, all other components are not autoclavable. Electronic systems (Fluigent) are perfused with 0.5% bleach, followed by 70% alcohol and sterile deionized water. The microfluidic device is decontaminated by UV and ozone treatment, and disposed after use. **b,** Priming of the different components of the five-condition platform is carried out under a class II biosafety cabinet. To prevent air accumulation in the fluidics, medium and drug bottle reservoirs and tubing are pre-filled by manually pushing air from 10 mL syringes into bottles closed with a cap having two ports. One medium bottle and the drug bottle are connected to the M-switch valve (Fluigent), in turn connected to the tubing with the capacitor. The second medium bottle is connected to a separate tubing with the second capacitor. Capacitors are 1 cm long Tygon tubing with an inner diameter of 2 mm and are connected via Luer fittings on each extremity. The whole tubing network is filled with medium. For loading bacteria, the inlet ports of the device are plugged with Tygon tubing through two metal connectors. The bacterial suspension contained in a syringe is loaded through the outlet port via a secondary tubing and leaving the primary outlet tubing clamped with a binder clip. Next, water is manually perfused into the inlet of the CL, after closing the CL outlet with a binder clip. Numbers indicate consecutive actions. **c,** Final assembly of the five-condition platform. After 10 min of incubation, the syringe containing the bacterial suspension is removed, and the secondary outlet tubing closed with a binder clip, disinfected, closed with a Luer lock and disinfected again. The bottle reservoirs are connected to the inlet ports of the device, and the outlet tubing is connected to the flowmeter (Fluigent) and to the sealed waste, after removing the binder clip. The system assembly is ready to be connected to the flow controller and to be mounted on the microscope stage.

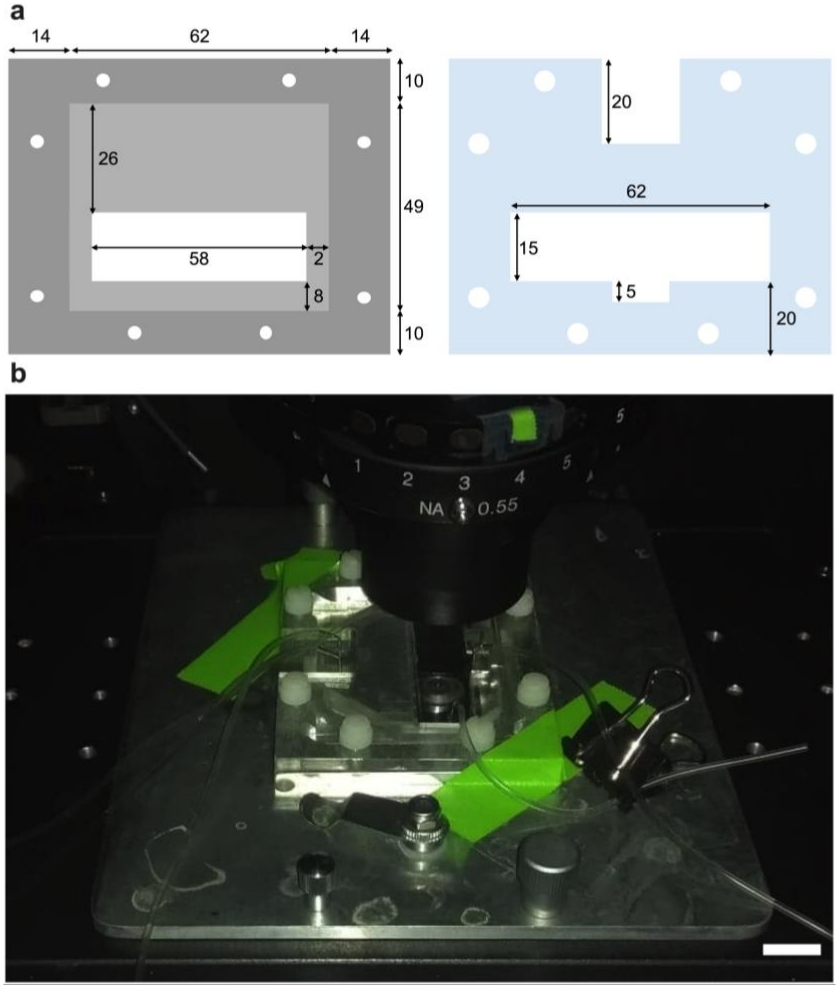

Supplementary Fig. 3

Mounting of the 5-condition device. a, Schematic of the metal and acrylic holders used to mount the five-condition device on the microscope stage. Circular holes are used to secure the system with plastic screws. Numbers indicate dimensions in millimeters. **b,** Picture of the microscope stage with the five-condition device fixed with metal clips and tape. Scale bar, 10 mm.

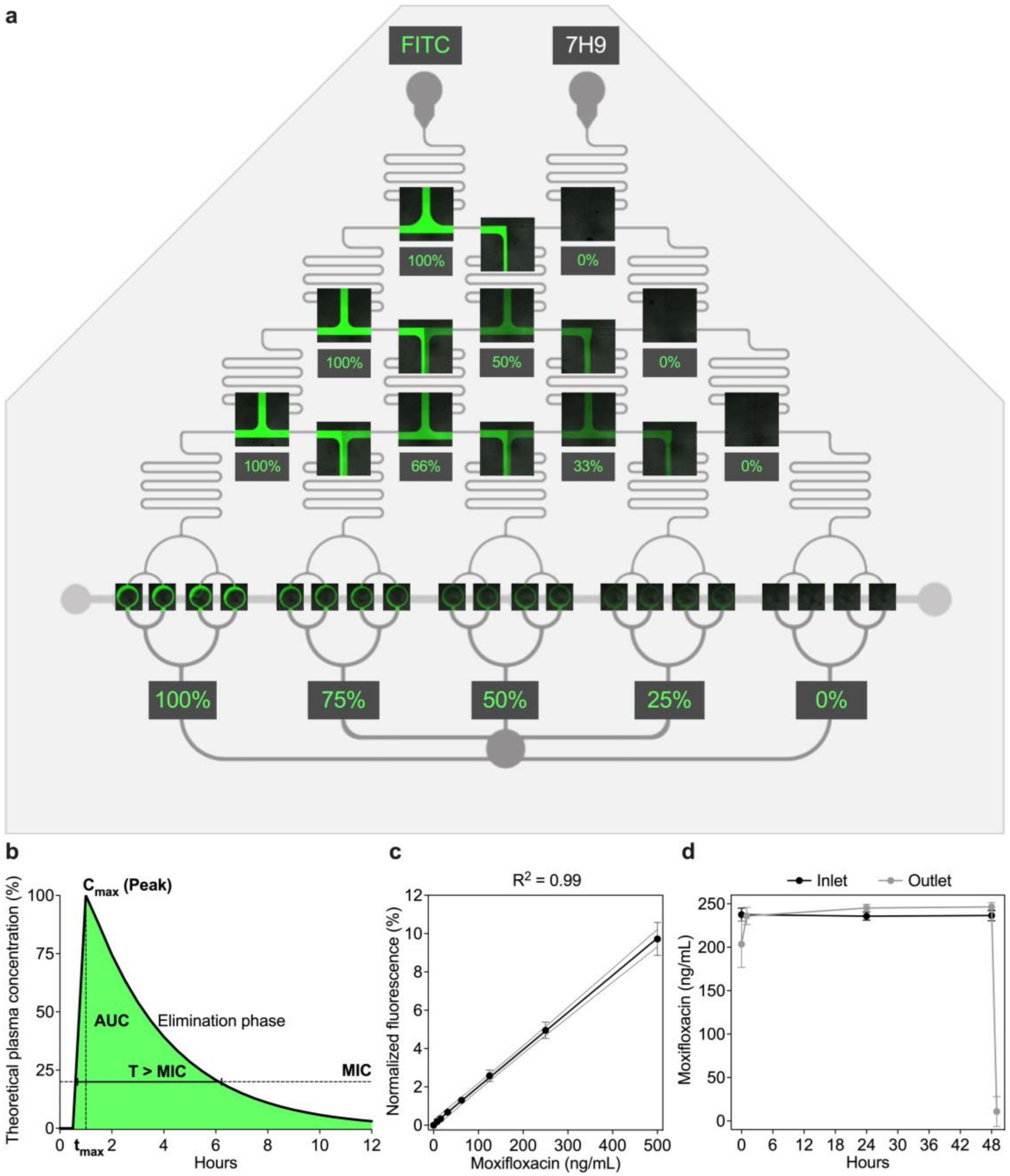

Supplementary Fig. 4

Generation of a static linear concentration gradient and moxifloxacin adsorption by PDMS. **a,** Representative images of serpentine junctions and exits and of the microchambers, overlaid on the plan of the 5-condition device, during steady-state injection of a FITC solution (100 µM) into the left inlet-port and of 7H9 into the right inlet-port, at a flow rate of 120 µL/h. The experiment was carried out three times. The dilution rates shown in the figure are the theoretical ones, derived from the assumption of a linear gradient. Bright field and fluorescence images are merged. Microchannels thickness, 0.3 mm, and microchambers diameter, 1 mm. **b,** Schematic of a theoretical drug PK profile, indicating MIC; peak plasma concentration (C_{max}); time to reach the

C_{max} (t_{max}); area under the curve (AUC); and time over the MIC (T > MIC). **c,** Calibration curve of moxifloxacin concentration in 7H9 medium by spectrofluorimetry. The contribution of 7H9 medium alone was subtracted from all fluorescence intensity datasets. Data are expressed as mean ± SD ($N = 3$). The relationship between moxifloxacin fluorescence intensity (Ex 287 nm, Em 465 nm) and concentration was found to be linear across the tested concentrations. Best-fit line and 99% confidence bands are also shown. **d,** Quantification of the concentration of moxifloxacin flowing in (black symbols and line) and out (gray symbols and line) of the 5-condition device. After 48 hours, 7H9 medium alone was perfused into the device, to confirm clearing of moxifloxacin from the system. Data are expressed as mean ± SD ($N = 4$).

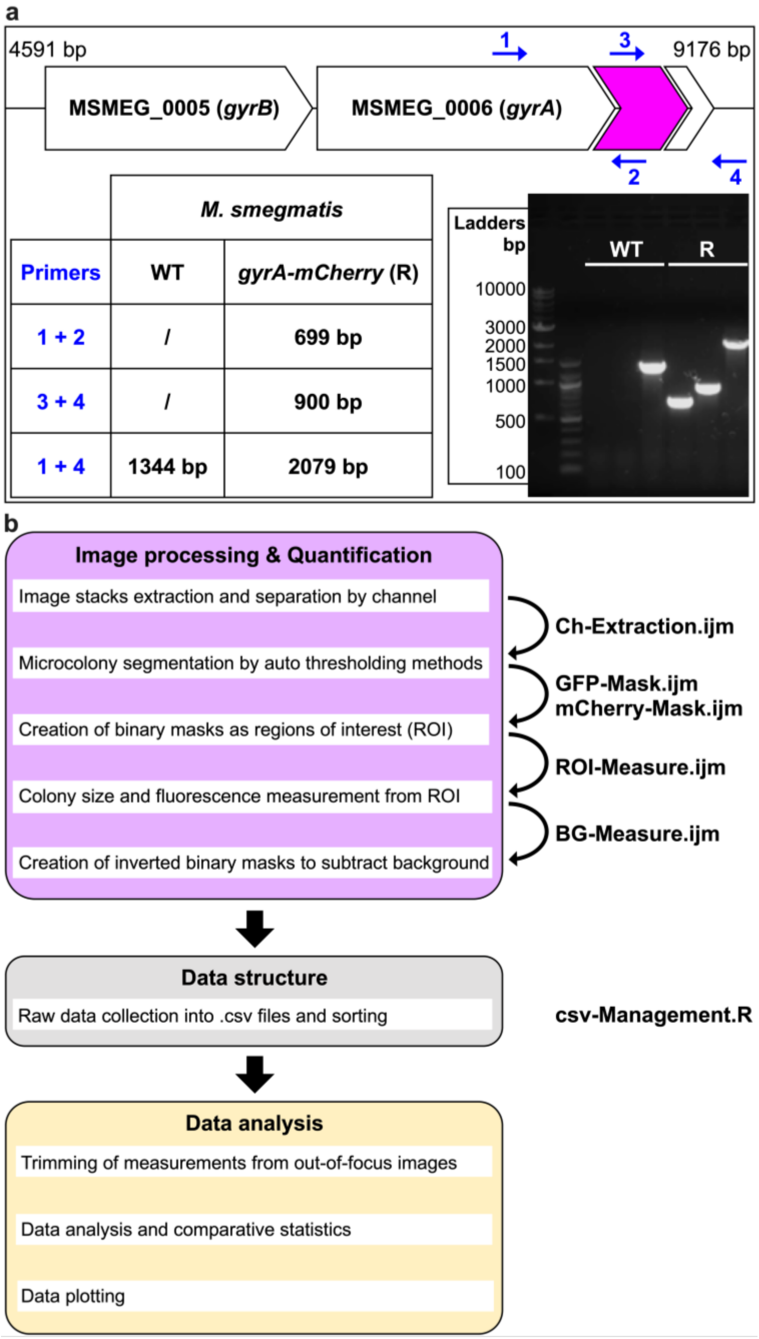

Supplementary Fig. 5

GyrA-mCherry reporter construction and image analysis workflow. a, Schematic of DNA gyrase encoding operon in *M. smegmatis* mc^{2}155 genome, with *mCherry* gene (magenta arrow) inserted in frame, just upstream of *gyrA* stop codon. Blue arrows represent the primers used for PCR analysis, to check *mCherry* chromosomal insertion. Expected amplicon sizes are indicated in the table for wild type (WT) and reporter (R) strains, and shown in a 1% agarose gel. **b,** Data analysis diagram separated into three steps: i. Extraction of image stacks from time-lapse movies, creation of masks and microcolony data extraction from ROI and inverted ROI (five ImageJ macros, lavender box); ii. Extraction of .csv files containing microcolony-derived parameters over

time (area, mean fluorescence and standard deviation of fluorescence) and corresponding background measurement for each image (one R script, gray box); iii. Removal of values from out-of-focus images, data processing, statistical analysis and data visualization (Excel and GraphPad Prism, yellow box).

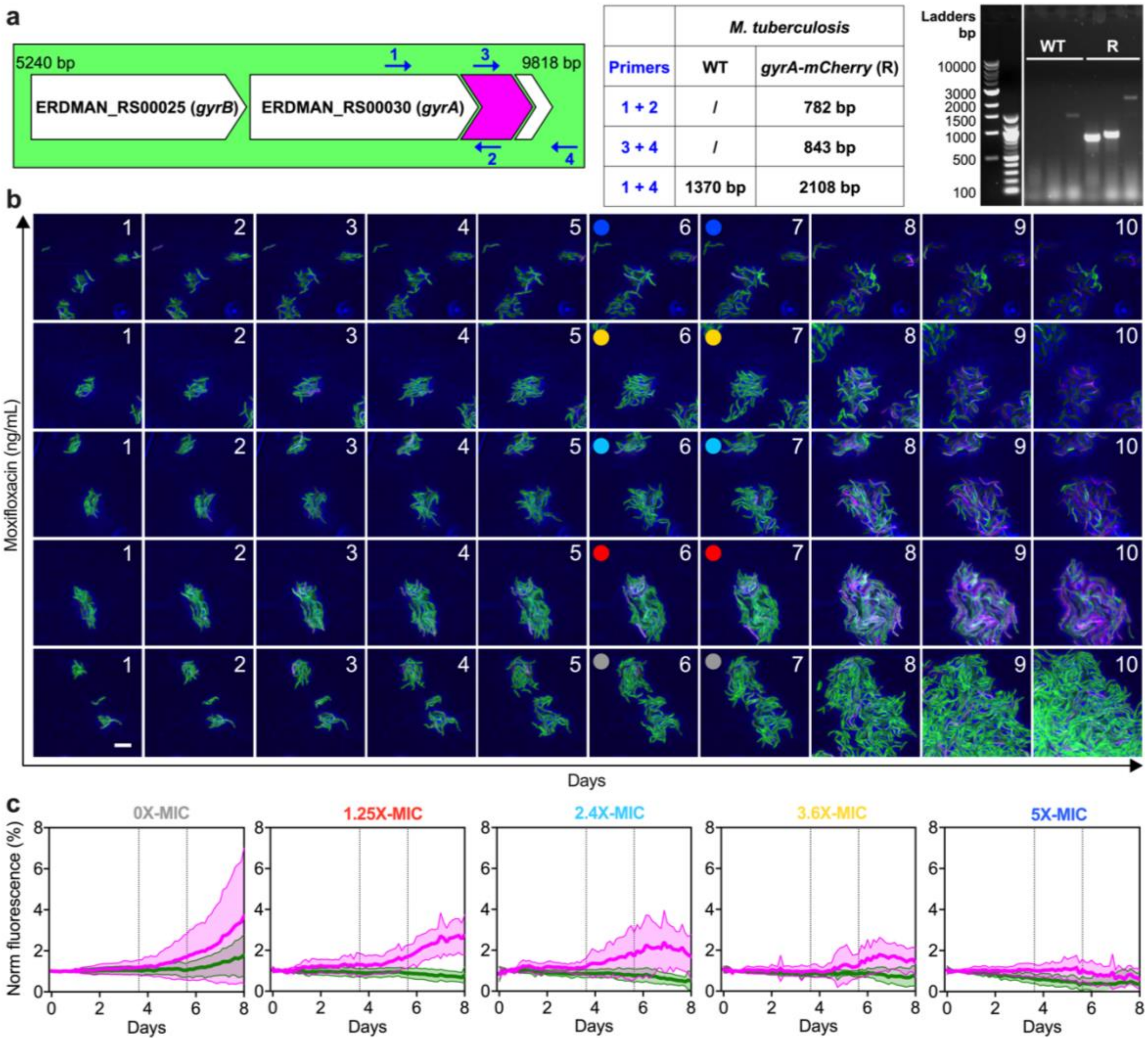

Supplementary Fig. 6

Multi-condition and multi-phasic time-lapse microscopy of *M. tuberculosis* GFP$_{cyt}$_GyrA-mCherry reporter. a, Schematic of DNA gyrase encoding operon in *M. tuberculosis* Erdman genome, with *mCherry* gene (magenta arrow) inserted in frame, just upstream of *gyrA* stop codon. Green color indicates constitutive expression of a green fluorescent marker in the cytosol (GFP$_{cyt}$). Blue arrows represent the primers used for PCR analysis, to check *mCherry* chromosomal insertion. Expected amplicon sizes are indicated in the table for wild type (WT) and reporter (R) strains, and shown in a 1% agarose gel. **b,** Time-lapse image series of exponentially growing *M. tuberculosis* seeded into the five-condition device and stressed with a stable gradient of moxifloxacin. Each row is representative of a chamber group. Colored circles indicate different moxifloxacin concentrations (gray: no drug; red: 62.5 ng/mL; light blue: 120 ng/mL; yellow: 180 ng/mL; blue: 250 ng/mL), according to the dilution factors measured in Fig. 3b. Phase-contrast and fluorescence channels are merged. Images were acquired every 3 hours and numbers represent days. Scale bar, 5 µm. **c,** Normalized microcolony GFP$_{cyt}$ (green) and GyrA-mCherry (magenta) fluorescence during time-lapse microscopy. Datasets are expressed as mean ± SD (13 < *N* < 45 microcolonies, from two independent experiments). Concentrations (within dotted lines) are relative to the MIC (50 ng/mL).

Supplementary Video Legends

Supplementary Video 1. PDMS membrane actuation. Representative movie of a microfluidic chamber, perfused with a FITC solution (100 µM), obtained with the ImageJ plugin Stack 3D Surface Plot. Fluorescence inside and outside the microchamber was acquired at constant flow in the FL (150 µL/h) and at incremental pressure steps in the CL and was represented by a heatmap as a function of the microchamber size. Decreasing fluorescence represents lowering of the PDMS membrane.

Supplementary Video 2. Combined time-lapse microscopy of *M. smegmatis* GyrA-mCherry reporter treated with a pulsing gradient of moxifloxacin. Representative movies of exponentially growing bacteria seeded into different microchambers of the 5-condition platform. Bacteria were first grown in fresh 7H9 medium for 6 hours, then one microchamber (left side) was left without drug and the other four microchambers were perfused with different pulsing concentrations of moxifloxacin (Moxi) for 12 hours. The peak concentrations are expressed relative to the MIC (50 ng/mL). Finally, fresh 7H9 medium was perfused everywhere for 6 hours. Images were recorded every 20 minutes (10 fps). GyrA-mCherry (magenta) and phase contrast (blue) channels are merged. Time in minutes and drug concentrations are indicated. Scale bar, 10 µm.

Supplementary Video 3. Combined time-lapse microscopy of *M. tuberculosis* GFP$_{cyt}$_GyrA-mCherry reporter treated with a static gradient of moxifloxacin. Representative movies of exponentially growing bacilli seeded into different microchambers of the 5-condition platform. Bacilli were first grown in fresh 7H9 medium for 5 days, then one microchamber (left side) was left without drug and the other four microchambers were perfused with different concentrations of moxifloxacin (Moxi) relative to the MIC (50 ng/mL) for 2 days. Finally, fresh 7H9 medium was perfused everywhere for 3 days. Images were recorded every 3 hours (10 fps). Constitutive GFP$_{cyt}$ (green), GyrA-mCherry (magenta) fluorescence and phase contrast (blue) channels are merged. Time in hours and drug concentrations are indicated. Scale bar, 5 µm.

Supplementary Table 1. Strains, plasmids and primers used in this study. Resistance markers are indicated and restriction sites are bolded.

REAGENT	SOURCE	IDENTIFIER
**Bacterial strains**		
Escherichia coli DH5α	Invitrogen	Cat#12297016
Escherichia coli TOP10	ThermoFisher	Cat#C404010
Mycobacterium smegmatis mc^{2}155	Lab collection	ATCC®700084
Mycobacterium tuberculosis Erdman	Lab collection	ATCC®35801
Mycobacterium smegmatis mc^{2}155_pGM235	This paper	GMS5
Mycobacterium tuberculosis Erdman_pGM234	This paper	GMT18
GMT18_pND235	This paper	GMT18-GFP
**Plasmids**		
pCR2.1-TOPO TA cloning plasmid, Amp^R, Km^R	Invitrogen	pCR2.1-TOPO
pJG1100 – Suicide vector expressing Km^R, Hyg^R resistance cassettes and *sacB* marker	Lab collection	pJG1100
pBS34, containing *mCherry*, Km^R	A gift from Eric Muller[86]	Addgene_83796
pJG1100-based vector for chromosomal knock-in of *linker-mCherry* in frame with *gyrA* (*rv0006*)	This paper	pGM234
pJG1100-based vector for chromosomal knock-in of *linker-mCherry* in frame with *gyrA* (*MSMEG_0006*)	This paper	pGM235
pND200 expressing GFP from UV15 strong promoter, Km^R	Lab collection	pND235
**Primers**		
Cloning, linker-*mCherry*-for<br>5'TG**CCTAGG**GGTAGCGGCAGCGGTAGCGTGAGCAAGG GCGAGGAGGATAAC3'	Microsynth	#1495386
Cloning, *mCherry*-rev<br>5'AT**GTTAAC**TTACTTGTACAGCTCGTCCATG CC3'	Microsynth	#1429770
Cloning, *MSMEG_0006* (*gyrA*) UPR-for<br>5'GC**TTAATTAA**TGCGGGAAGGCGACGAACTGG3'	Microsynth	#1676562
Cloning, *MSMEG_0006* (*gyrA*) UPR-rev<br>5'AG**CCTAGG**CGCCTCGGGTGACTCGGCGGT3'	Microsynth	#1676563
Cloning, *MSMEG_0006* (*gyrA*) DNR-for<br>5'CT**GTTAAC**TGGAAACGCTGAGGTCCCCGA3'	Microsynth	#1676564
Cloning, *MSMEG_0006* (*gyrA*) DNR-rev<br>5'AT**GGCGCGCC**ACGCCACGGCGATCATCCATA3'	Microsynth	#1676565

CT1_KI_Msm_for<br>5’GACTTCGACTCCAACCGCTCC3’	Microsynth	#1692255
CT2_mCherry_rev<br>5’CATGTGCACCTTGAAGCGCAT3’	Microsynth	#1692256
CT3_mCherry_for<br>5’ACCACCTACAAGGCCAAGAAG3’	Microsynth	#1692257
CT4_KI_Msm_rev<br>5’TTGACCAGGCCGATGAGGGCC3’	Microsynth	#1692258
Cloning, *ERDMAN_RS00030* (*gyrA*) UPR-for<br>5’GC**TTAATTAA**GAGCTGGTCGGTGCGGTGCTG3’	Microsynth	#1707232
Cloning, *ERDMAN_RS00030* (*gyrA*) UPR-rev<br>5’AG**CCTAGG**ATTGCCCGTCTGGTCTGCGCC3’	Microsynth	#1707233
Cloning, *ERDMAN_RS00030* (*gyrA*) DNR-for<br>5’CT**GTTAAC**TCAGGCTCGCCCGACGACGATGCGGATC3’	Microsynth	#1721329
Cloning, *ERDMAN_RS00030* (*gyrA*) DNR-rev<br>5’AT**GGCGCGCC**CTCTCGATCCCGCGCCACCCTCCGCGG<br>G3’	Microsynth	#1721330
CT1_KI_Mtb_for<br>5’TCGCCCAGGTCATCCAGATTC3’	Microsynth	#1824597
CT4_KI_Mtb_rev<br>5’GGATCTGCATGCTGGCTCGAA3’	Microsynth	#1824598

Supplementary Data

Supplementary Data 1. AutoCAD mask design of 5-condition platform. FL (up) and CL (down) are shown.

Supplementary Data 2. Ch-Extraction.ijm. ImageJ macro for channels extraction from image stacks.

Supplementary Data 3. GFP-Mask.ijm. ImageJ macro to generate binary masks from green fluorescence.

Supplementary Data 4. mCherry-Mask.ijm. ImageJ macro to generate binary masks from red fluorescence.

Supplementary Data 5. ROI-Measure.ijm. ImageJ macro to quantify different parameters from segmented images.

Supplementary Data 6. BG-Measure.ijm. ImageJ macro to quantify the background fluorescence.

Supplementary Data 7. csv-Management.R. R Studio script to sort datasets for analysis.
